# Killing of melanoma cells and their metastases by human lactoferricin derivatives requires interaction with the cancer marker phosphatidylserine

**DOI:** 10.1007/s10534-014-9749-0

**Published:** 2014-05-18

**Authors:** Sabrina Riedl, Beate Rinner, Helmut Schaider, Karl Lohner, Dagmar Zweytick

**Affiliations:** 1 Biophysics Division, Institute of Molecular Biosciences, University of Graz, Schmiedlstraße 6, 8042 Graz, Austria; 2 Center for Medical Research, Medical University of Graz, Stiftingtalstraße 24, 8010 Graz, Austria; 3 Cancer Biology Unit, Department of Dermatology, Medical University of Graz, Auenbruggerplatz 8, 8036 Graz, Austria; 4 Southern Clinical Division, Dermatology Research Centre, School of Medicine, The University of Queensland, Ipswich Road 33, Woolloongabba, QLD 4102 Australia

**Keywords:** Peptides, Cancer therapy, Liposomes, Melanoma, Membrane biophysics, Lactoferricin derivatives, Cell membrane permeabilization, Phosphatidylserine

## Abstract

Despite favorable advancements in therapy cancer is still not curative in many cases, which is often due to inadequate specificity for tumor cells. In this study derivatives of a short cationic peptide derived from the human host defense peptide lactoferricin were optimized in their selective toxicity towards cancer cells. We proved that the target of these peptides is the negatively charged membrane lipid phosphatidylserine (PS), specifically exposed on the surface of cancer cells. We have studied the membrane interaction of three peptides namely LF11-322, its *N*-acyl derivative 6-methyloctanoyl-LF11-322 and its retro repeat derivative R(etro)-DIM-P-LF11-322 with liposomes mimicking cancerous and non-cancerous cell membranes composed of PS and phosphatidylcholine (PC), respectively. Calorimetric and permeability studies showed that *N*-acylation and even more the repeat derivative of LF11-322 leads to strongly improved interaction with the cancer mimic PS, whereas only the *N*-acyl derivative also slightly affects PC. Tryptophan fluorescence of selective peptide R-DIM-P-LF11-322 revealed specific peptide penetration into the PS membrane interface and circular dichroism showed change of its secondary structure by increase of proportion of β-sheets just in the presence of the cancer mimic. Data correlated with in vitro studies with cell lines of human melanomas, their metastases and melanocytes, revealing R-DIM-P-LF11-322 to exhibit strongly increased specificity for cancer cells. This indicates the need of high affinity to the target PS, a minimum length and net positive charge, an adequate but moderate hydrophobicity, and capability of adoption of a defined structure exclusively in presence of the target membrane for high antitumor activity.

## Introduction

In 2008 more than 12.7 million people worldwide were newly diagnosed with cancer accounting for 7.6 million deaths (http://www.who.int/mediacentre/factsheets/fs297/en/). Prospects of the World Health Organization (WHO) suggest a further increase of new cases in 2030 to 11 million deaths. Even though in the last decades, much progress has been achieved regarding chemotherapy, surgery, radiotherapy, targeted therapy and combinations thereof there are still many disadvantages and problems that have to be handled. These are e.g., severe side effects due to non-adequate specificity for tumor cells or drug resistance.

A promising strategy is the development of new cancer therapies in the form of host defense peptides (Hoskin and Ramamoorthy 2008; Papo and Shai 2005; Riedl et al. 2011b) of various origins and derivatives thereof. Host defense peptides are mostly cationic amphipathic peptides, targeting the cell membrane by electrostatic interactions with anionic molecules at the cell surface (Riedl et al. 2011b). Though some different types of anionic molecules are provided by cancer cells that could account for the selectivity of these membrane active peptides towards cancer cells (Riedl et al. 2011b) a major target discussed is the negatively charged phospholipid phosphatidylserine (PS), which is reported to be exposed on the outer leaflet of the cancer cell membrane (Connor et al. 1989; Ran et al. 2002; Riedl et al. 2011a; Schröder-Borm et al. 2005; Utsugi et al. 1991; Zweytick et al. 2011a). In non-cancerous cells the outer leaflet of the plasma membrane exhibits an overall neutral charge due to its main components: the zwitterionic phosphatidylcholine (PC) and sphingomyelin (SM), whereas PS together with phosphatidylethanolamine (PE) only assembles the inner leaflet (Bevers et al. 1996). This regularly occurring asymmetric distribution of the major phospholipids between the two membrane leaflets is well documented (Bevers et al. 1998; Zwaal and Schroit 1997) and is normally maintained by an ATP-dependent-aminophospholipid translocase (Seigneuret and Devaux 1984) and—floppase (Zwaal and Schroit 1997). Further activation of a third protein, the lipid scramblase, by influx of Ca^2+^ into the cytoplasm is reported to cause a rapid transbilayer phospholipid mixing leading to a nearly symmetric distribution across the membrane bilayer (Zwaal and Schroit 1997). Thus, cellular changes like depletion of ATP and/or influx of Ca^2+^ into the cytoplasm seem to be important factors accounting for exposure of PS by cancer cells. Furthermore, as proposed by Ran et al. (Ran et al. 2002) injury and activation of tumor endothelium by cytokines and reactive oxygen species might induce PS exposure of tumor vessels. Also from lipid analytic studies in our laboratory with melanoma and metastases thereof we saw that not the overall PS content of cancer cells increases compared to non-cancer cells, but seemingly only the asymmetric PS distribution in the plasma membrane gets lost (Riedl et al. 2011a).

Already in 1991 Utsugi et al. (1991) reported about elevated expression of PS in the outer leaflet of human tumor cells. In 2002 Ran et al. (2002) reported about increased exposure of anionic phospholipids specifically on the surface of tumor blood vessels. Our recent studies strongly supported these findings and demonstrated that exposure of PS to the outer leaflet of membranes is a general phenomenon for cancer plasma membranes independently on cancer type and is also characteristic for metastases and many cancer types including those with poor outcome or treatability, like malignant melanoma (Riedl et al. 2011a). PS exposure would normally lead to recognition by macrophages (Fadok et al. 1992) and dentritic cells (Martin et al. 1995) and consequently start of apoptosis which can however be circumvented by cancer cells by different ways (Miyashita and Reed 1993; Soengas et al. 2001). Importantly we have also proven that cancer specific PS exposure was no sign of apoptosis and not only true for cancer cell lines but also for primary cancer cell cultures (Riedl et al. 2011a). This yields a promising overall marker for cancer as well as a specific target for host defense peptides (Riedl et al. 2011a).

In the present study, we focused on the human host defense peptide lactoferricin (LFcin) which is reported to exhibit antimicrobial, antiviral, anti-inflammatory and antitumor activities (Bezault et al. 1994; Gifford et al. 2005; Vogel et al. 2002). Human lactoferricin comprises amino acid residues 1–45 of the N-terminus of human Lactoferrin (hLF). LF11, an 11 amino acid fragment of hLFcin has already been optimized regarding its activity against bacterial membranes (Brandenburg et al. 2010; Zweytick et al. 2006, 2008, 2011b) and since bacterial as cancer membranes comprise negatively charged phospholipids on their surface and antimicrobially active peptides often also possess antitumor activity (Wang et al. 2009) it was reasonable to test LF11 derivatives, as LF11-322 and others exhibiting antimicrobial activity (Zweytick et al. 2011b) with cancer cell mimics and cancer cells. One peptide family was selected for thorough investigations: LF11-322, its *N*-acylated derivative 6-MO-LF11-322 (6-methyloctanoyl-LF11-322) and its “(retro) repeat”-derivative R-DIM-P-LF11-322 (LF11-322-proline-LF11-322 retro). LF11-322 is an LF11-derivative exhibiting good antibacterial activities and it has been shown in previous antibacterial studies that *N*-acylation enhances the antibacterial activity due to the higher hydrophobic potency (Zweytick et al. 2006, 2011b). Since it was reported by Yang et al. (2004) that for a potent antitumor peptide a minimum length and minimum net positive charge are needed for appropriate antitumor activity, R-DIM-P-LF11-322 has been designed to fulfill these requirements. The addition of the retro sequence to the peptide moiety LF11-322 separated by a short spacer (1 proline) aimed to create a sequence composed of the peptide and its mirror image to increase amphipathicity and hence antitumor activity. In this article an insight in the structural and mechanistic requirements for a selective antitumor peptide like R-DIM-P-LF11-322 is given. The peptide exhibited potent activity against the cancer cell lines of melanoma (SBcl-2) and melanoma metastases (WM164) that were previously reported to expose PS (Riedl et al. 2011a), whereas for the non-cancer cell lines such as melanocytes lacking PS exposure (Riedl et al. 2011a) no toxicity appeared.

## Materials and methods

### Materials

1,2-dipalmitoyl-*sn*-glycero-3-phosphocholine (DPPC), 1-palmitoyl-2-oleoyl-*sn*-glycero-3-phosphocholine (POPC), 1,2-dipalmitoyl-*sn*-glycero-3-phospho-l-serine (Na-salt) (DPPS) and 1-palmitoyl-2-oleoyl-*sn*-glycero-3-phospho-l-serine (Na-salt) (POPS) were purchased from Avanti Polar Lipids, Inc. (USA), and used without further purification. Stock solutions of DPPC and POPC were prepared in CHCl_3_/CH_3_OH (2:1, v/v), stock solutions of DPPS and POPS were prepared in CHCl_3_/CH_3_OH (9:1, v/v) and stored at −18 °C. The amidated peptides LF11-322 (PFWRIRIRR-NH_2_, M = 1,298.6 g/mol), its *N*-6-methyloctanoyl derivative 6-MO-LF11-322 (CH_3_CH_2_–CH_2_(CH_3_)–(CH_2_)_4_–CO–NH-PFWRIRIRR-NH_2_, M = 1,438.9 g/mol) and R-DIM-P-LF11-322 (PFWRIRIRRPRRIRIRWFP-NH_2_, M = 2,677.4 g/mol) were purchased from NeoMPS, Inc. (San Diego, CA, USA). The purities were >96 % as determined by RP-HPLC. Peptides were dissolved in Acetic acid (0.1 %, v/v) at a concentration of 3 mg/ml. Peptide solutions were stored at 4 °C and concentrations were determined by measurement of UV-absorbance of tryptophan at 280 nm. ANTS (8-aminonaphthalene-1,3,6-trisulfonic acid, disodium salt) and DPX (*p*-xylene-bis-pyridinium bromide) used for permeability studies were purchased from Molecular Probes (Eugene, OR). Sodiumdodecylsulfate (SDS) and dodecylphosphocholine (DPC) used for CD measurements were purchased from Carl Roth GmbH&Co (Karlsruhe, Germany) and Avanti Polar Lipids, Inc. (USA), respectively.

### Cell lines and culture

The melanocytic cell lines were kindly provided by Dr. Meenhard Herlyn (The Wistar Institute, Philadelphia, PA). Melanoma cell lines from primary (SBcl-2) and metastatic (WM164) lesions were grown in RPMI 1640 medium with stable l-glutamine (Invitrogen, UK), supplemented with 2 % FBS. Human melanocytes used as healthy control cells were isolated from foreskin. Foreskin was cut into small pieces and incubated with 0.3 % trypsin (PAA) overnight at 4 °C and for 1 h at 37 °C. Epidermis was separated. Cells were mechanically removed from the cell layer and centrifuged at 300 g for 3 min. The pellet was re-suspended and cells further cultured in melanocyte growth medium (Biomedica, Vienna, Austria, PromoCell GmbH, Heidelberg, Germany). All cells were kept in a 5 % CO_2_ atmosphere at 37 °C. At 90 % confluence cell-culture flasks were passaged after detachment with accutase (PAA, Pasching Austria). All cell cultures were periodically checked for mycoplasma.

### Toxicity studies: propidium iodide-uptake assay

Cells were collected, re-suspended in media and diluted to a concentration of 10^6^ cells/ml. Aliquots of 10^5^ cells/100 µl media were incubated with different amount of peptides (0–80 µM) for 8 h at 37 °C and 5 % CO_2_. Propidium iodide (PI) (2 µl/10^5^cells of 50 µg/ml, Invitrogen, Camarillo, CA, USA) was added and cells were again incubated for 5 min at room temperature in the dark. Excitation and emission wavelengths were 536 and 617 nm, respectively. Cytotoxicity was calculated from the percentage of PI positive cells in media alone (P_0_) and in the presence of peptide (P_X_). (see Eq. 1). Triton-X-100 was used to determine 100 % of PI positive cells (P_100_).

1 $$\% PI - uptake = \frac{{100*(P_{X} - P_{0} )}}{{(P_{100} - P_{0} )}}$$

### Preparation of liposomes

Appropriate amounts of respective phospholipid stock solution were dried under a stream of nitrogen and stored in vacuum overnight to completely remove organic solvents. The dry lipid film was then dispersed in phosphate buffered saline (PBS, 20 mM NaPi, 130 mM NaCl, pH 7.4) and hydrated at a temperature well above the gel to fluid phase transition of the respective phospholipid under intermittent vigorous vortex-mixing. The lipid concentration was 0.1 weight % for calorimetric and 2 % for leakage experiments. Hydration was carried out in presence or absence of peptides at a lipid-to-peptide ratio of 25:1 and 12.5:1 using a protocol described for POPS (Jiménez-Monreal et al. 1998), DPPS (Jing et al. 2005) and DPPC (Sevcsik et al. 2007). Briefly hydration of POPS films occurred at 30 °C, DPPS at 65 °C and DPPC at 50 °C, for 2 h with vortexing for 1 min every 15 min. The hydration of the lipid mixtures DPPS/DPPC 1:5 and DPPS/DPPC 1:1 were performed at 65 °C for 2 h by vortexing accompanied by one freeze–thaw cycle every 15 min. The fully hydrated samples were stored for at least 1 h at room temperature until measurement.

### Differential scanning calorimetry (DSC)

DSC experiments were performed with a differential scanning calorimeter (VP-DSC) from MicroCal, Inc. (Northhampton, MA, USA). Heating scans were performed at a scan rate of 30 °C/h (pre-scan thermostating 30 min) with a final temperature of approximately 10 °C above the main transition temperature (T_m_) and cooling scans at the same scan rate (pre-scan thermostating 1 min) with a final temperature approximately 20 °C below T_m_. The heating/cooling cycle was performed three times. Enthalpies were calculated by integration of the peak areas after normalization to phospholipid concentration and baseline adjustment using the MicroCal Origin software (VP-DSC version). The phase transition temperature was defined as the temperature at the peak maximum (McElhaney 1982).

### Fluorescence spectroscopy

Fluorescence spectroscopy experiments were performed using a SPEX Fluoro Max-3 spectrofluorimeter (Jobin–Yvon, Longjumeaum, France) and spectra were analyzed with Datamax software.

### Tryptophan quenching

Tryptophan fluorescence spectra were obtained at room temperature using an excitation wavelength of 282 nm and a slit width of 5 nm for excitation and emission monochromators. Quenching of Tryptophan was carried out in the presence and absence of phospholipid liposomes (lipid-to-peptide ratio 25:1) using 0.1, 0.4 and 0.7 M acrylamide. The data were analyzed according to the Stern–Volmer equation (Eq. 2):

2 $${{F_{0} } \mathord{\left/ {\vphantom {{F_{0} } F}} \right. \kern-0pt} F} = 1 + K_{SV} \left[ Q \right]$$

where F_0_ and F represent the fluorescence emission intensities in the absence and presence of the quencher molecule (Q) and K_SV_ is the Stern–Volmer quenching constant, which is a quantitative measure for the accessibility of tryptophan to acrylamide (Tao and Cho 1979).

### ANTS/DPX leakage experiments

Leakage of aqueous contents from liposomes was determined using the 8-aminonaphthalene-1,3,6-trisulfonic acid/*p*-xylene-bis-pyridinium bromide (ANTS/DPX) assay (Ellens et al. 1985). Lipid films (preparation see above) were hydrated with 12.5 mM ANTS, 45 mM DPX, 68 mM NaCl, 10 mM HEPES (4-(2-hydroxyethyl)-1-piperazineethanesulfonic acid) at pH 7.4 following a standard procedure. Subsequently, the dispersions were extruded 20 times through a polycarbonate filter (Millipore-Isopore™) of 0.1 µm pore size to obtain LUVs. Unilamellarity and size were tested by X-ray and dynamic light scattering, respectively. The ANTS/DPX encapsulating vesicles were separated from free ANTS/DPX by exclusion chromatography using a column filled with Sephadex™ G-75 (Amesham Biosciences) fine gel swollen in an isosmotic buffer (10 mM HEPES, 140 mM NaCl, pH 7.4). The void volume fractions were collected and the phospholipid concentration was determined by phosphate analysis (Bartlett 1959; Broekhuyse 2005). The fluorescence measurements were performed in 2 mL of the isosmotic buffer in a quartz cuvette at 37 °C. Aliquots of LUVs were diluted with the isosmotic buffer to a final lipid concentration of 50 µM. Fluorescence spectra were obtained at 37 °C using an excitation wavelength of 360 nm, an emission wavelength of 530 nm and a slit width of 5 nm for both excitation and emission monochromators. Fluorescence emission was recorded as a function of time before and after the addition of incremental amounts of peptide. The fluorescence increase due to leakage and subsequent dilution of quenched dye was measured after addition of peptides. Peptides were added to final concentrations of 2, 4 and 8 µM, corresponding to peptide-to-lipid molar ratios of 1:25, 1:12.5 and 1:6.25, respectively. Data are presented in terms of fluorescence intensity (I_F_ and were calculated using Eq. 3:

3 $$I_{F} = \frac{{F - F_{0} }}{{F_{\hbox{max} } - F_{0} }}$$

F is the measured fluorescence, F_0_ the initial fluorescence without peptide and F_max_ the fluorescence corresponding to 100 % leakage gained by addition of 1 % Triton X-100.

### Circular dichroism (CD) spectroscopy

Measurements were performed on a Jasco J 715 Spectropolarimeter (Jasco, Gross-Umstadt, Germany) at room temperature using quartz cuvettes with an optical path length of 0.02 cm. The CD spectra were measured between 260 and 180 nm with a 0.2 nm step resolution. To improve accuracy 5 scans were averaged. Peptides were dissolved in 10 mM Hepes (pH 7.4) to a final concentration of 100 µM. Spectra were measured in the absence and presence of 1 mM sodium dodecyl sulfate (SDS) or 1 mM dodecylphosphocholine (DPC) mimicking cancer and healthy mammalian membranes, respectively. The respective peptide-to-surfactant molar ratios were 1:25 and 1:100. Background signals were subtracted after measurements. Percentage secondary structure calculations were done using Dichroweb, CDSSR Convolution Program using reference set 4 (Whitmore and Wallace 2004, 2008).

### PEP-FOLD: de novo peptide structure prediction

The peptide secondary structures and folding were predicted by PEP-FOLD, an online resource for de novo peptide structure prediction. PEP-FOLD provides pdb (protein data bank) files with representation of macromolecular structure. Pdb files were visualized by DSViewer Pro (Accelrys Software Inc.).

## Results

In this study, we report on activity, respectively selectivity of human lactoferricin derivatives for melanoma and melanoma metastases over differentiated non-tumorigenic counterparts as melanocytes in vitro and in model systems. The for toxicity studies used cell lines of melanoma (SBcl2) and melanoma metastases (WM164) were recently shown to expose the negatively charged lipid PS, whereas the non-tumorigenic melanocytes, were shown to lack PS exposure (Riedl et al. 2011a). The three peptides studied LF11-322, 6-MO-LF11-322 and R-DIM-P-LF11-322 (for primary sequence see Materials and Table 1) were originally derived from the membrane active peptide LF11 (FQWQRNIRKVR-NH_2_) (Zweytick et al. 2006, 2011b).

**Table 1:** Amino acid (aa) sequence, – number (no.) and net charge of peptides LF11-322, 6-MO-LF11-322 and R-DIM-P-LF11-322

Peptide	Amino acid sequence	No. of aa/net charge
LF11-322	PFWRIRIRR-NH_2_	9/+5
6-MO-LF11-322	6-MO-PFWRIRIRR-NH_2_	9/+4
R-DIM-P-LF11-322	PFWRIRIRR P RRIRIRWFP-NH_2_	19/+9

### Cancer toxicity and selectivity in vitro

**Fig. 1:**
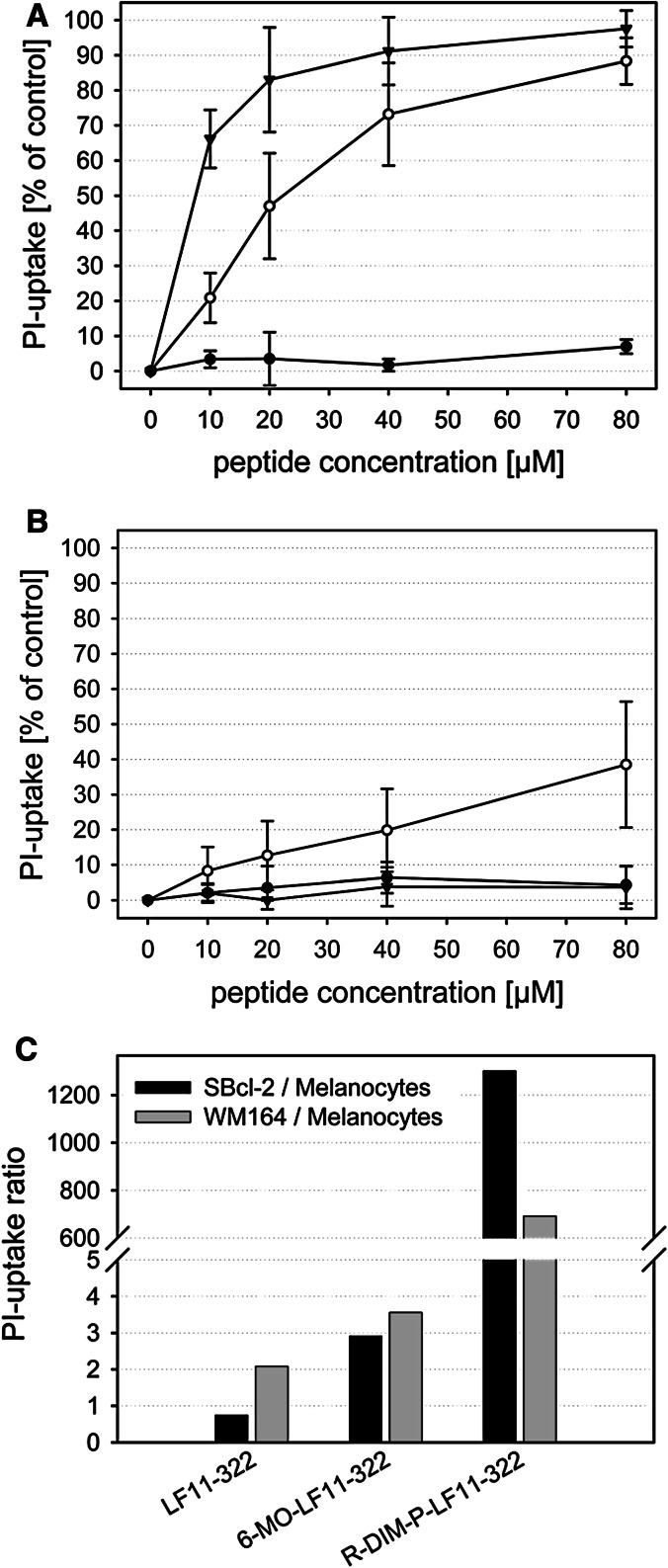
Peptide toxicity/PI-uptake of cancer and non-cancer cell lines. **a** Concentration dependent cytotoxic activity of LF11-322 (*filled circle*), 6-MO-LF11-322 (*open circle*) and R-DIM-P-LF11-322 (*triangle*) against melanoma cell line SBcl-2 after 8 h of incubation with peptides; **b** Concentration dependent cytotoxic activity against primary cultures of differentiated non-tumorigenic melanocytes after 8 h of incubation with peptides; **c** specificity of peptides at 20 µM peptide concentration after 8 h of incubation displayed as PI-uptake ratio of melanoma SBcl-2 versus melanocytes and melanoma metastases WM164 versus melanocytes

Cytotoxic activity of the peptides towards melanoma cells of primary (SBcl-2) and metastatic lesions (WM164) and differentiated non-tumorigenic melanocytes was determined by measurement of PI-uptake, which only occurs when integrity of the cell membrane is lost. Cells were incubated in media containing serum for 8 h in the presence of peptides. Peptide concentrations were varied from 0 to 80 µM. Fig. 1a, b illustrate that LF11-322 is only minor active against the melanoma cell line SBcl-2 with <10 % killing at a peptide concentration of 80 µM, as well as against melanocytes with a moderate twofold selectivity for WM164 cells at 20 µM peptide concentration (Fig. 1C). *N*-Acylation in the case of 6-MO-LF11-322 significantly improves cancer cell toxicity. Nevertheless at its IC_50_ of ~20 µM peptide concentration it already kills ~15 % of the non-cancer melanocytes, again yielding a rather modest threefold to fourfold specificity for cancer over non-cancer cells (Fig. 1c). Interestingly the specificity of 6-MO-LF11-322 for cancer cells is sevenfold after 1 h (data not shown) but subsequently decreases to threefold to fourfold after 8 h (Fig. 1c). The repeat peptide R-DIM-P-LF11-322 is even more active against SBcl-2 than the *N*-acylated peptide 6-MO-LF11-322, but with increased specificity for cancer cells. Already at a peptide concentrations of 20 µM, R-DIM-P-LF11-322 yields more than 80 % PI positive SBcl-2 cells, while only <1 % of differentiated non-tumorigenic melanocytes are killed, exhibiting a specificity higher than 1,000-fold for cancer cells at this peptide concentration (see Fig. 1c). The melanoma cell line of metastatic lesions, WM164, tested at 20 µM R-DIM-P-LF11-322 peptide concentration is also highly sensitive to the peptide (specificity >500-fold) (Fig. 1c).

### Activity and selectivity in model systems: mechanistic and structural studies

Based on the fact that cancer membranes specifically expose the negatively charged lipid PS (Riedl et al. 2011a; Utsugi et al. 1991) inclusively the peptide sensitive tumor cell lines SBcl2 and WM164 (Riedl et al. 2011a), different liposomal mimics of human cancerous and non-cancerous cell membranes were applied to study the respective membrane interaction of peptides: DPPS and POPS, negatively charged phospholipids exposed on the outer leaflet of plasma membranes of cancer cells, and DPPC or POPC, a zwitterionic phospholipid mimicking plasma membranes of non-neoplastic cells. For circular dichroism studies SDS and DPC were used as cancer or non-cancer models, respectively.

### Differential scanning calorimetry: destabilization of cancer and non-cancer model membranes

**Fig. 2:**
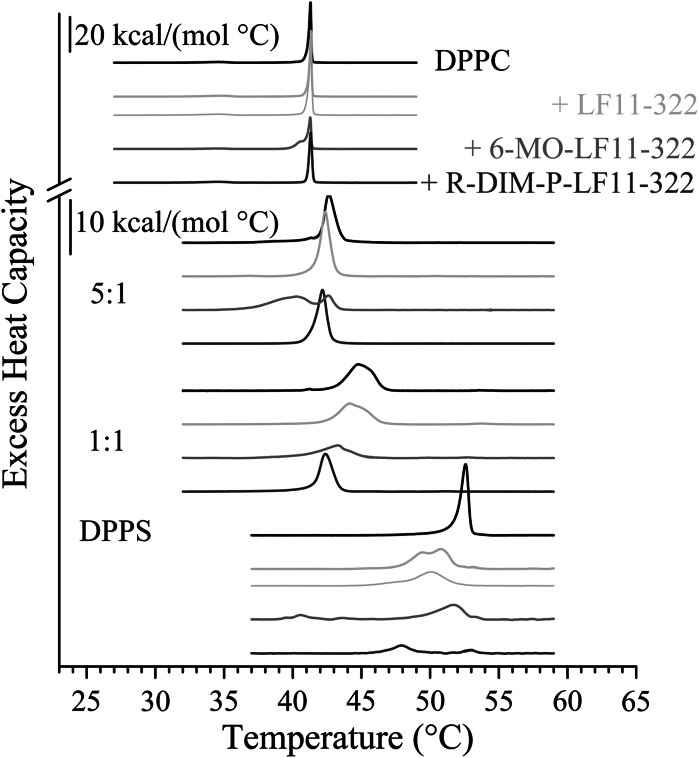
DSC thermograms of DPPC, DPPC/DPPS 1:5 (molar ratio), DPPC/DPPS 1:1 (molar ratio) and DPPS in the absence and presence of LF11-322 (*light gray*), 6-MO-LF11-322 (*gray*) and R-DIM-P-LF11-322 (*dark gray*) (25:1, lipid-to-peptide molar ratio *thick lines*, 12:1, lipid-to-peptide molar ratio *thin lines*). For clarity, the DSC curves were displayed on the ordinate by an arbitrary increment

Consistently with published data (Lewis and McElhaney 2000), the temperature-dependence of the access heat capacity of pure DPPS shows one phase transition (Fig. 2 bottom) which corresponds to the main chain-melting from the lamellar-gel (*L*_β_) to the liquid-crystalline (*L*_α_) phase at 52.6 °C. Addition of LF11-322, 6-MO-LF11-322 and R-DIM-P-LF11-322 (25:1 lipid-to-peptide molar ratio) results in a decrease of the main-transition enthalpy (Δ*H*_m_) of DPPS, but to different extent: DSC thermograms show that LF11-322 only slightly reduces the main transition enthalpy of PS by 10 %. However, N-acylation such as 6-MO-LF11-322 has a stronger effect leading to a reduction of the transition enthalpy by 35 %. Obviously the repeat sequence, in form of R-DIM-P-LF11-322, leads to the most severe perturbance of the cancer mimic DPPS, indicated by a strong decrease of the transition enthalpy by 70 % (see Table 2). In the presence of all peptides the main-transition temperature (*T*_m_) shifts to lower values, due to a destabilization of the gel phase. Again the repeat sequence exhibits the by far strongest effect by a decrease of the transition temperature by nearly 5 °C and a broad peak over the whole transition range. The thermogram of the LF11-322 treated liposome is split into at least two peaks due to different peptide affected lipid domains, where the lower temperature transition is presumably more highly enriched in peptide, a peptide effect of the non-acylated LF11-322 also observed with the bacterial model system phosphatidylglycerol (Zweytick et al. 2011b). Addition of the *N*-acylated peptide 6-MO-LF11-322 however causes a main peak at decreased transition temperature and a smaller transition at 40.6 °C, the latter most likely cannot be related to the lamellar gel to liquid crystalline transition. This is confirmed by the wide-angle X-ray (WAXS) pattern (data not shown) of DPPS liposomes in presence of 6-MO-LF11-322 which shows existence of a small fraction of fluid hydrocarbon chains already at 25 °C, which is the same at 46 °C. At 46 °C however WAXS indicates less order of hydrocarbon chains in the presence than in the absence of the lipopeptide. This is again in line with DSC results showing a decreased transition enthalpy in presence of this peptide and a decreased main transition temperature suggesting destabilization of the gel phase. The decrease of cooperativity indicated by an increase of the transition half-width (Δ*T*_1/2_) (Table 2) shown for all 3 endotherms is due to differentially severe loss of cooperativity. Comparable results were observed for POPS liposomes (data not shown). Importantly, the effect of the repeat (double) sequence R-DIM-P-LF11 is much higher than the effect of the single sequence LF11-322 at doubled concentration (+LF11-322; 12.5:1 lipid-to-peptide ratio; Fig. 2, thin line), excluding a simple concentration effect. This is in correlation with the toxicity studies showing a much higher than double cancer toxicity of R-DIM-P-LF11-322 comparing with LF11-322.

**Table 2:** Thermodynamic parameters of DDPS (cancer cell mimic), DPPC (non-cancer cell mimic), and 1:5 and 1:1 (molar ratios) mixtures thereof in the absence and presence of LF11-322, 6-MO-LF11-322 and R-DIM-P-LF11-322 at a lipid to peptide molar ratio of 25:1 (unless otherwise noted) (ad Fig. 2)

	Δ*H*_pre_ (kcal/mol)	T_pre_ (°C)	Δ*H*_m_ (kcal/mol)	T_m_ (°C)	T_1/2_ (°C)
DPPC	1.0	34.6	9.1	41.3	0.19
+LF11-322	1.1	34.8	9.0	41.3	0.17
+LF11-322 (12.5:1)	1.0	34.5	9.2	41.3	0.26
+6-MO-LF11-322	0.4	34.6	9.1	(40.7)/41.3	0.26
+R-DIM-P-LF11-322	1.5	34.5	9.1	41.3	0.25
DPPC/DPPS 5:1	–	–	9.0	42.6	0.89
+LF11-322	–	–	10.1	42.4	0.80
+6-MO-LF11-322	–	–	7.2/1.6	42.2/42.6	3.30/1
+R-DIM-P-LF11-322	–	–	8.2	42.2	0.83
DPPC/DPPS 1:1	–	–	9.8	44.8	2.21
+LF11-322	–	–	8.3	44.2	2.42
+6-MO-LF11-322	–	–	5.4	43.3	2.45
+R-DIM-P-LF11-322	–	–	7.9	42.4	1.08
DPPS	–	–	9.9	52.6	0.65
+LF11-322	–	–	4.7/4.5	49.3/50.9	1.7/1.4
+LF11-322 (12.5:1)	–	–	2.1/4.9	47.8/50.1	2.48
+6-MO-LF11-322	–	–	(0.7)^a^/5.9	(40.6)^a^/51.7	2.24
+R-DIM-P-LF11-322	–	–	3.1	47.9	1.53

^a^Not related to main transition

Unlike DPPS and POPS thermograms of DPPC exhibit two transitions (Fig. 2 top) as well in agreement with published data (Koynova and Caffrey 1998). The transition at 34.6 °C corresponds to the pre-transition, from the lamellar-gel (*L*_β_′) to the ripple-phase (*P*_β_′). The second transition can be attributed to the main-transition at 41.3 °C (from the ripple-phase (*P*_β_′) to the liquid-crystalline (*L*_α_) phase). In contrast to DPPS and POPS, DPPC liposomes remain unperturbed by addition of LF11-322, even when the concentration of peptide is doubled (see Fig. 2; Table 2) (LF11-322; 12.5:1 lipid-to-peptide molar ratio) or upon addition of the repeat form R-DIM-P-LF11-322, where only a slight loss of cooperativity occurs indicated by a minor increase of the half width of the transition. This goes in hand with the non-toxic effect of these peptides on non-tumor cells, such as melanocytes. Addition of 6-MO-LF11-322 to DPPC liposomes (25:1 lipid-to-peptide molar ratio) however results in some perturbance of DPPC membranes displayed by a shoulder at decreased transition temperature with decreased cooperativity (Table 2).

We also investigated by DSC the effect of the peptides on liposomes composed of mixtures of DPPC/DPPS (1:5 and 1:1, molar ratios). In the presence of peptides in both mixtures the phase transitions are shifted to lower temperatures closer to the lower melting component DPPC (41.3 °C), indicating a preferential interaction with the component DPPS. This is most significant in presence of 6-MO- and R-DIM-P-LF11-322. However the *N*-acylated peptide in the mixture with only 20 % DPPS (80 % DPPC) already induced a strong broadening and splitting of the transition that implies an interaction with both lipid components. The peptide R-DIM-P-LF11-322 in both mixtures induces a significant thinning of the transition at reduced temperatures and reduced enthalpies suggesting a strong but specific interaction with DPPS only.

### ANTS/DPX leakage: membrane permeabilization

The membrane permeabilizing effect of peptides (2–8 µM) on cancer and non-cancer mimics was also tested by induction of leakage of ANTS/DPX from large unilamellar vesicles composed of POPS and POPC, respectively.

**Fig. 3:**
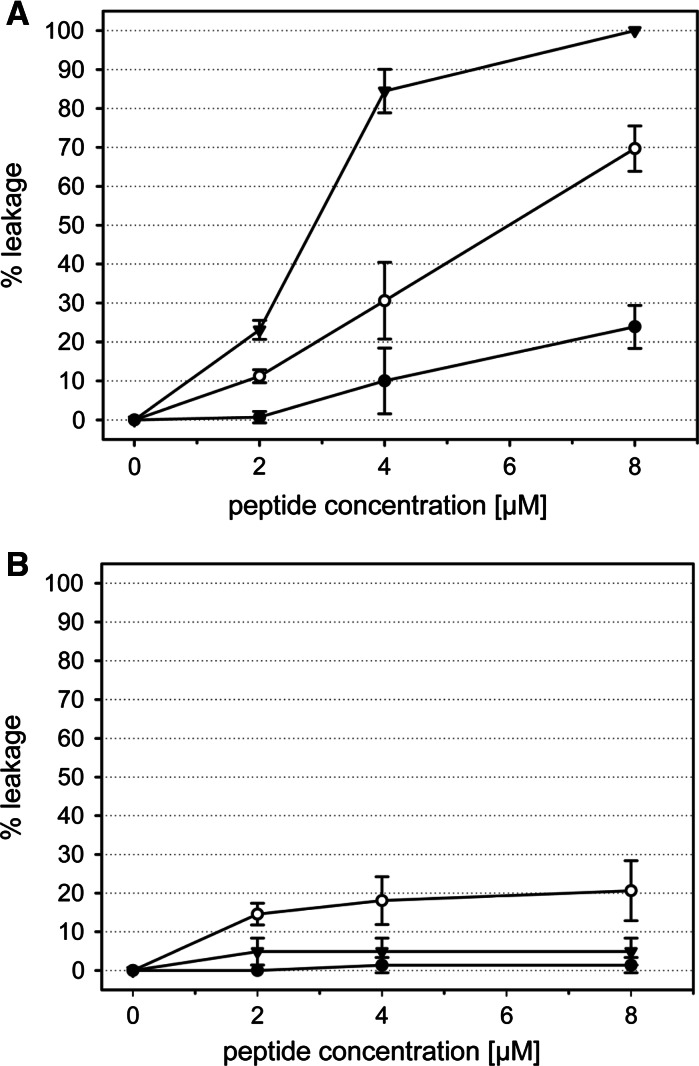
ANTS/DPX leakage of LUVs composed of POPS (**a**) and POPC (**b**) as a function of the concentration of LF11-322 (*filled circle*), 6-MO-LF11-322 (*open circle*) and R-DIM-P-LF11-322 (*triangle*). Concentration of LUVs was 50 µM and temperature was kept at 37 °C during measurements. Complete lysis was determined by addition of Triton X-100 and zero levels correspond to fluorescence before peptide addition

As illustrated in Fig. 3a, addition of the different peptides to POPS vesicles, mimicking cancer cell membranes, leads to diverse release of ANTS/DPX. N-acylation increases the weak ANTS/DPX release induced by 4 and 8 µM LF11-322 (~20 % at highest concentration 8 µM) by a factor of three up to ~30 and 70 %, respectively. Whereas the repeat sequence R-DIM-P-LF11-322 is most effective in terms of permeability increase at all concentrations inducing leakage up to 90–100 % at e.g. highest peptide concentration.

Fig. 3B demonstrates ANTS/DPX release from pure POPC liposomes, mimicking non-cancerous cell membranes. Obviously, addition of LF11-322 and R-DIM-P-LF11-322 does not result in any significant release of the fluorophore (<5 %). Only the *N*-acylated peptide 6-MO-LF11-322 provokes also slight leakage of POPC vesicles of about 20 % at a peptide-to-lipid molar ratio of 1:6.25, providing only threefold specificity for the cancer mimic. The repeat sequence R-DIM-P-LF11-322 exhibits the highest activity for the cancer mimic with up to 20-fold selectivity for cancer over non-cancer mimics being again in good correlation with its high specificity for cancer cells in vitro.

### Tryptophan quenching: peptide solubility and penetration depth

Trp-emission properties like emission wavelength and fluorescence quenching by acrylamide can be used to study changes in solubility of peptides and localization of Trp in different environments, as in solution or in the presence of membranes composed of different lipids. The emission spectra of the 1 Trp of LF11-322, 6-MO-LF11-322 and the 2 Trp of R-DIM-P-LF11-322 in buffer exhibit a maximum (λ_em,max_) near 356 nm (see Table 3). This is in agreement with the emission maximum of Trp exposed to polar environment as e.g. to solvent. Together with the high Stern–Volmer constant of LF11-322, 6-MO-LF11-322 and R-DIM-P-LF11-322 (K_SV_ ~ 20 M^−1^) in solution an absence of peptide aggregation can be assumed. By comparing Trp fluorescence in solution with fluorescence in the presence of the cancer mimics POPS and DPPS (lipid-to-peptide molar ratio 25:1) it is obvious that the Stern–Volmer constant (K_SV_) is dramatically decreased by all peptides tested with strongest effect of the R-DIM-P-LF11-322. This observation gives evidence for a proportionally less polar environment of the Trp of the three peptides in the presence of the cancer mimic PS indicating localization at the interface of the PS bilayer or deeper. Additionally, a significant blue shift of the emission wavelength could be observed for LF11-322, 6-MO-LF11-322 and R-DIM-P-LF11-322 giving as well evidence of penetration of the Trp into the membrane interface. The K_SV_ for all peptides is lower in the presence of anionic phospholipids with unsaturated (POPS) than saturated (DPPS) fatty acyl chains, which seems feasible since peptides can penetrate more easily in the less tightly packed membranes of POPS. Besides acyl chains of POPS are fluid at the experimental temperature, whereas the acyl chains of DPPS are still in the gel phase. The repeat sequence R-DIM-P-LF11-322 shows strongest changes in characteristics in both systems indicating deepest penetration of the peptides tested into the hydrophobic core of the cancer membrane mimicking bilayer.

**Table 3:** Stern-Volmer quenching constant (K_SV_) and maxima of emission wavelength (λ_em,max_) of LF11-322, 6-MO-LF11-322 and R-DIM-P-LF11-322 in PBS, POPS, DPPS and DPPC membranes at a lipid to peptide ratio of 25:1

Peptide	K_SV_ in PBS (M^−1^) (λ_em,max_) (nm)	K_SV_ in POPS (M^−1^) (λ_em,max_) (nm)	K_SV_ in DPPS (M^−1^) (λ_em,max_) (nm)	K_SV_ in DPPC (M^−1^) (λ_em,max_) (nm)
LF11-322	19.7 (354)	5.4 (338)	7.9 (334)	8.8 (354)
6-MO-LF11-322	24.5 (353)	4.5 (342)	7.0 (337)	10.5 (344)
R-DIM-P-LF11-322	19.1 (353)	4.1 (334)	4.3 (335)	13.2 (352)

In agreement with the other model studies no effect (no pronounced blueshift) was seen with peptides LF11-322 and R-DIM-P-LF11-322 in the presence of DPPC, the non-cancerous cell mimic, which implies no membrane penetration of Trp in this mimic. Only 6-MO-LF11-322 also exhibits a blue shift in the presence of DPPC by 9 nm, indicating less specific membrane interaction than by the other peptides.

### Circular dichroism spectroscopy and PEP-FOLD: secondary structure vs. activity and selectivity

**Fig. 4:**
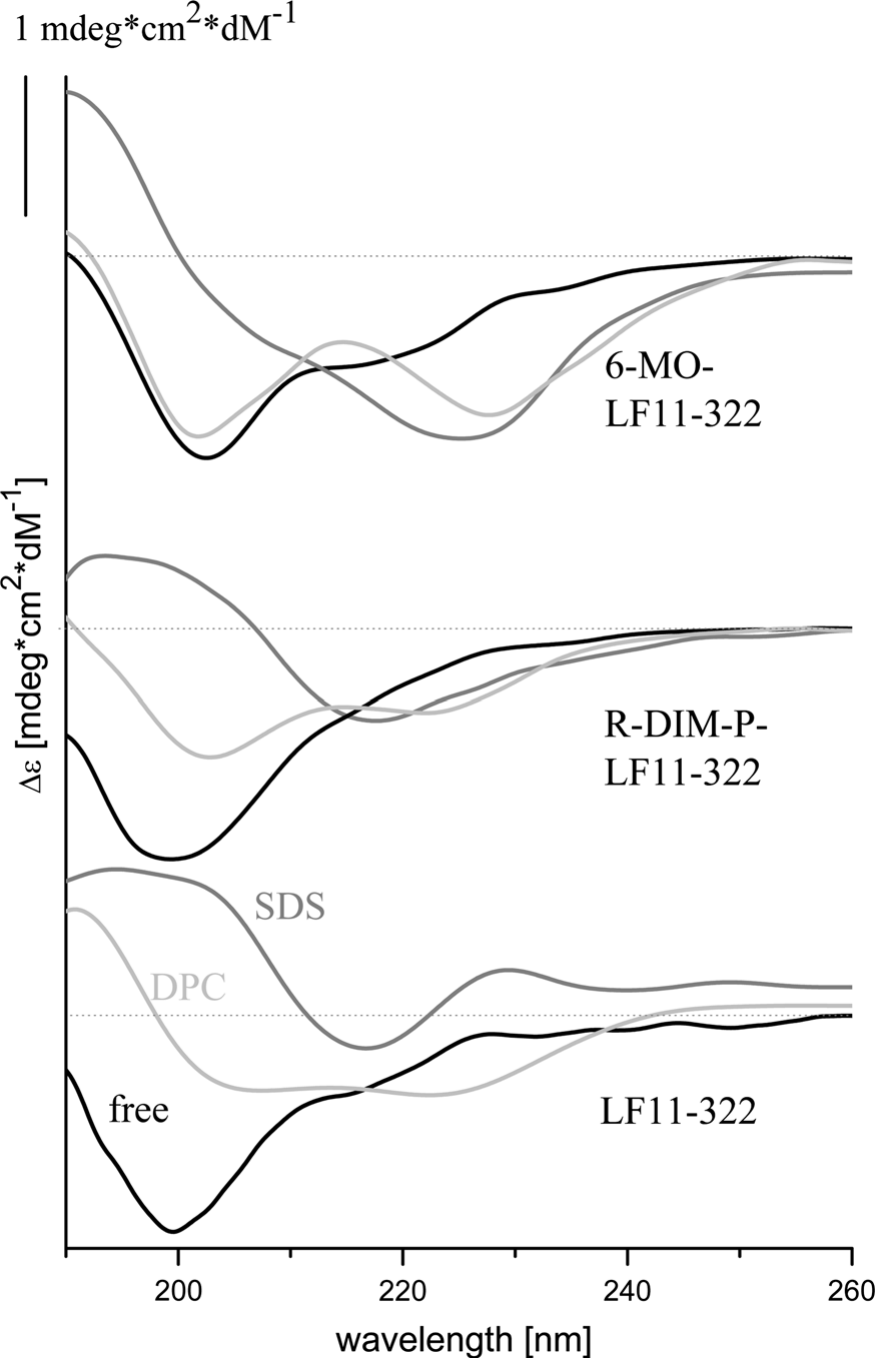
CD spectra of LF11-322, R-DIM-P-LF11-322 and 6-MO-LF11-322 in the presence of buffer (*black*), the non-cancer mimic DPC (*light grey*) and the cancer mimic SDS (*grey*)

**Fig. 5:**
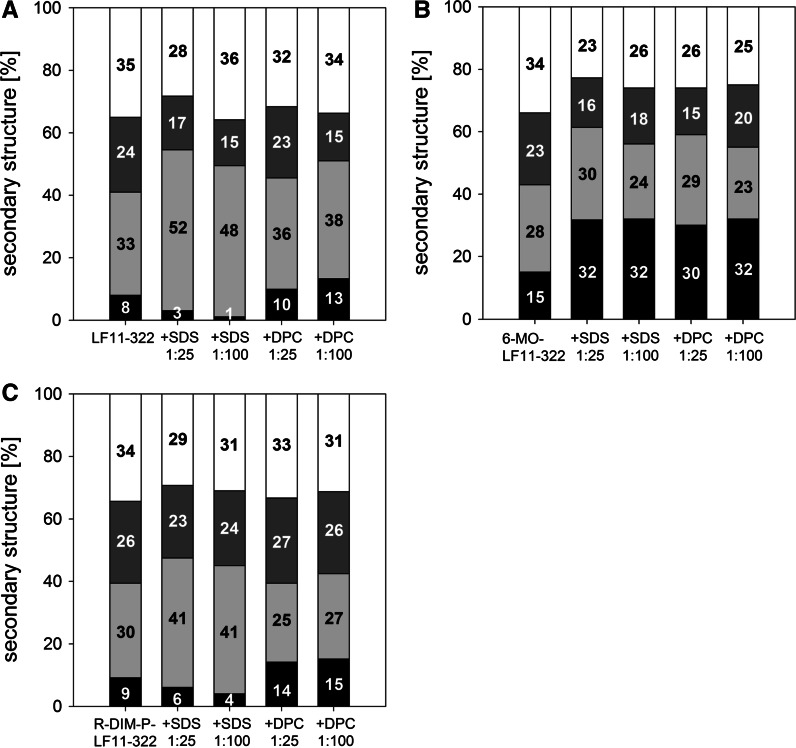
Secondary structure distributions. Analysis of CD spectra of free peptides LF11-322, 6-MO-LF11-322 and R-DIM-P-LF11-322 in solution and in complex with SDS and DPC micelles. Distribution of secondary structures of LF11-322 (**a**), 6-MO-LF11-322 (**b**) and R-DIM-P-LF11-322 (**c**) in Hepes buffer (*first bar*) or presence of SDS and DPC at peptide to surfactant ratios of 1:25 and 1:100 were calculated using Dichroweb, CDSSR Convolution Program using reference set 4 (*27;28*). The α-helical content is shown in *black* at the *bottom*; β-sheet in *light grey*; turns in *dark grey*; random coil structures in white at the *top*

By circular dichroism studies significant differences in secondary structure of the three peptides in lipidic environment were observed (Fig. 4). Only in solution all peptides exhibit similar structural properties, being mainly unstructured and showing β-sheet conformation and turns to similar extent (analyzed data Fig. 5a–c). By 15 % the *N*-acylated peptide (Fig. 5b) exhibits the highest though still minor proportion of α-helical content in solution. Since CD-experiments in lipidic environment as POPS or POPC deliver rather noisy data, SDS was taken as negatively charged cancer and DPC as neutral non-cancer mimic. Two different peptide-to-lipid ratios were used, 1:25 to define conditions comparable to model studies, 1:100 to ensure complete binding of whole peptide amount, rather comparable to conditions in vitro. Nevertheless only LF11-322 (Fig. 5a) in the presence of SDS shows some enhanced effect at higher peptide concentration. The other peptides do not show any significant structural changes at different concentrations, therefore it can be assumed that also at high peptide-to-lipid ratios no or all peptide is bound. Strikingly the selective peptides LF11-322 (Fig. 5a) and R-DIM-P-LF11-322 (Fig. 5c) exhibit a significant increase of β-sheet conformation in the presence of the cancer mimic SDS, the α-helical content is even further decreased. Moreover the structure of these peptides in the presence of the healthy mimic DPC is comparable to structure in solution, giving hint for a structure dependent cancer selective toxicity of these peptides. Strikingly only the less selective peptide 6-MO-LF11-322 exhibits a significant increase of the α-helical content to same extent in the presence of both cancer and non-cancer mimic. The presence of SDS enhances a positive band at 230 nm exhibited by LF11-322 (Fig. 4).

**Fig. 6:**
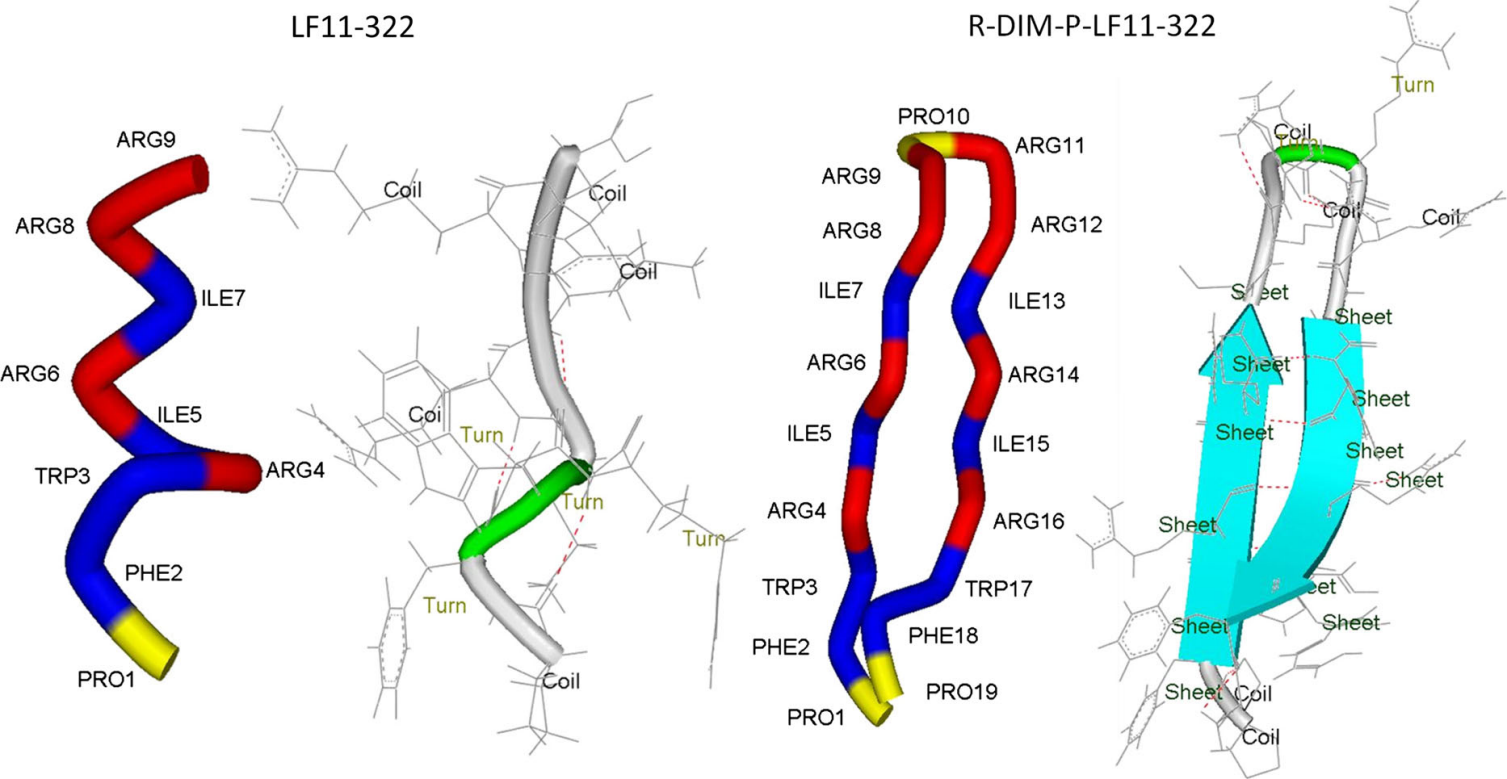
Secondary structure predictions for peptide LF11-322 and R-DIM-P-LF11-322 were performed by use of the program PEP-FOLD (Maupetit et al. 2009, 2010; Thévenet et al. 2012). The best model was plotted with DS ViewerPro 5.0 for Windows. On the particular *left sides* the amino acids are colored corresponding to their hydrophobicity according to the whole residue (octanol) hydrophobicity scale by Wimley and White (Wimley and White 1996). (hydrophilic amino acids are colored in *red*, hydrophobic amino acids are colored in *blue*, slightly charged amino acid Pro is colored in *yellow*). On the particular *right sides* predicted secondary structures are plotted and delineated, predicted hydrogen bonds are indicated as *red dashed lines*

As can be seen in Fig. 6 the structure prediction performed by the program PEP-FOLD (Maupetit et al. 2009, 2010; Thévenet et al. 2012) (http://bioserv.rpbs.univ-paris-diderot.fr/PEP-FOLD/) reveals a β-sheet conformation for R-DIM-P-LF11-322, which is conform with CD spectra of the peptide in presence of SDS. For the short peptide LF11-322, no α-helix or β-sheet structure is predicted, probably because the amount of only 9 amino acids is too low for formation of such structures. Two turns between Phe^2^ and Arg^6^ are proposed. It is also nicely demonstrated that the β-sheet structure of R-DIM-P-LF11-322 can be stabilized by formation of several possible hydrogen bonds (H-bonds) (red dashed lines in Fig. 6).

## Discussion

It has been shown that plasma membranes of cancer cells selectively expose the negatively charged lipid PS (Riedl et al. 2011a; Utsugi et al. 1991), which offers a potent target for amphipathic cationic host defense peptides (Papo and Shai 2005; Riedl et al. 2011b). In the present study, we could ascribe the selective antitumor activity of human lactoferricin derivatives to the selective activity against the cancer cell mimic PS (Table 4). A lipid rather than a receptor target would be advantageous, to minimize the risk of generation of resistance by mutagenesis upon application.

**Table 4:** Correlation of activity exhibited by peptides LF11-322, 6-MO-LF11-322 and R-DIM-P-LF11-322 in model and in in vitro studies

Peptide	LF11-322	6-MO-LF11-322	R-DIM-P-LF11-322
Amino acid sequence	PFWRIRIRR-NH_2_	6-MO-	PRRIRIRWFP-NH_2_
Net charge	+5	+4	+9
Hydrophobicity—∆G_woct_	5.64	3.34	7.12
Cancer mimic/healthy mimic			
Bilayer perturbation—DSC	+/−	++/+	+++/−
Permeability—ANTS/DPX leakage	+/−	++/+	+++/−
Bilayer affinity—quenching	++/−	++/+	++/−
Main structure—CD	β-sheet/as in solution	α-helical/α-helical	β-sheet/as in solution
Cancer cells/healthy cells			
Toxicity—PI uptake	−/−	++/+	+++/−
Cancer specificity	(+)	+ −	++++

For calculation of ∆G_woct_ of 6-MO-LF11-322 instead of the 6-MO residue N-acetylation was taken

^a^∆G_woct_ [kcal/mol] Peptide hydrophobicity expressed as transfer free energy of peptides from water to n-octanol (∆G_woct_) calculated from the whole-residue hydrophobicity scale taking in account the contribution of the C-terminal amide (Wimley and White 1996)

+, peptide effect; ++, increasing peptide effect; etc.; −, no peptide effect

As shown in this study the non-acylated peptide LF11-322 exhibited only weak activity against melanoma cancer cell lines, whereas the *N*-acylated derivative 6-MO-LF11-322 showed elevated and the repeat sequence R-DIM-P-LF11-322 highest activity of peptides studied. Only 6-MO-LF11-322 exhibited also moderate toxicity for non-cancer melanocytes though at respective elevated concentrations. So derivative R-DIM-P-LF11-322 emerged to be a peptide highly active and specific for cancer cells. Propidium iodide-uptake of melanoma cells upon incubation with peptides 6-MO-LF11-322 and R-DIM-P-LF11-322 further nicely demonstrates that the peptides might operate via a membrane mediated way, since propidium iodide can only be taken up by cells that suffer membrane disintegration. Actually host defense peptides are mainly described to act via a non-receptor mediated pathway against the target cell membrane by membranolytic effects; they can either trigger necrosis or apoptosis of cancer cells (Papo and Shai 2005; Riedl et al. 2011b). For triggering necrosis they directly kill by disrupting the target plasma membrane, whereas for triggering apoptosis they have to selectively enter cancer cells and cause mitochondrial swelling with consequent release of cytochrome c (Papo and Shai 2005). Bovine lactoferricin, e.g. was shown to selectively induce apoptosis in human leukemia and carcinoma cell lines (Mader et al. 2005).

The correlation of activity against bacteria with activity on bacterial model systems for lactoferricin derived peptides and their *N*-acylated derivatives was shown recently (Zweytick et al. 2011b). However, as reported increase of activity of LF11-322 by *N*-acylation as 6-MO-LF11-322 on the bacterial model system did not completely correlate with increase in antimicrobial activity, which seems to be due to the fact that elevated binding of *N*-acylated peptides to lipopolysaccharides (LPS) of the outer membrane of Gram-negative bacteria counteracts the elevated membrane permeabilizing potency on the cytoplasmic membrane (Zweytick et al. 2011b).). In this study however we demonstrate that antitumor activity as well as activity on the tumor model system PS is improved by N-acylation to a similar extent. Therefore it can be speculated that PS is a main target of the peptide on human cancer membranes and peptides are not reduced in their effective concentration by other membrane components, like LPS on bacteria. 6-MO-LF11-322, however did not only exhibit elevated activity against the cancer model but also to some extent against the non-cancer model PC correlating with moderate toxicity also against non-cancer melanocytes. A fact that goes conform with elevated hemolytic activity already described for *N*-acylated derivatives of LF11 as 6-MO-LF11-322 (20 % lysis of 2.5 % RBCs at 500 µg peptide/ml, (Zweytick et al. 2011b)) or C12LF11 (85 % lysis of 0.25 % RBCs at 100 µg peptide/ml (Zweytick et al. 2006)). Lower specificity might be due to increased peptide hydrophobicity by *N*-acylation indicated by a decrease of transfer free energy (∆G_woct_) of peptides from water to *n*-octanol (Wimley and White 1996), from 5.64 to 3.34 kcal/mol (see Table 4).

However, the repeat derivative of LF11-322, R-DIM-P-LF11-322, exhibits even less hydrophobicity (∆G_woct_ 7.12 kcal/mol) than LF11-322, but highly improved activity against cancer cell lines without loss of selectivity. It has been reported by Yang et al. (Yang et al. 2004) that a prerequisite for antitumor but not for antibacterial peptides derived of bovine lactoferrin is a minimum net charge close to +7 and to consist of at least 14 amino acids. And really, LF11-322 (9 amino acids; net charge +5) exhibited considerable antibacterial activity with a minimal inhibitory concentration for *E. coli* of 8–16 µg/ml (Zweytick et al. 2011b) but only negligible antitumor activity. We therefore designed R-DIM-P-LF11-322 (19 amino acids; net charge +9) a peptide composed of the peptide moiety LF11-322 linked to itself retro sequence via peptide bond over the amino acid Pro as a linker. The peptide moiety sequence was taken as template because it was not toxic for normal cells and the retro sequence added with a Pro linker should further help to stabilize the peptide structure via hydrogen bonds. The idea of addition of the retro sequence is to design a “twin”-peptide with increased amphipathicity. And actually cancer-toxicity is highly increased, in fact much more than doubled, by the repeat sequence peptide and like shown for the *N*-acyl derivative improved interaction of R-DIM-P-LF11-322 with the cancer mimic PS correlates with increased activity against the melanoma cancer cell line. Also no interaction with the healthy mimic PC correlates with no toxicity against non-cancer cells. R-DIM-P-LF11-322 seems to exhibit a high membrane destabilization effect together with highly increased induction of membrane permeability of PS bilayers. Besides, permeability studies show that a certain threshold concentration of the peptide is needed for induction of sufficient leakage of ANTS/DPX, differentiating it from highly lytic but mostly unspecific peptides like melittin, which induces leakage already at very low concentrations (data not shown) (Papo and Shai 2005). As mentioned, by model studies, such as calorimetry or ANTS/DPX leakage and in vitro studies it could be demonstrated that the effect of the repeat sequence peptide is even much higher than that of the peptide moiety at double concentration, excluding a simple mass and charge effect. As shown in model studies with mixtures of DPPC and DPPS the peptide R-DIM-P-LF11-322 seems to be able to act on the cancer membrane by specific clustering of the PS component, indicated by a strong decrease of T_m_ and T_1/2_ of the mixture, a mechanism also described for antimicrobial peptides as gramicidin S, magainin, PGLa and others (Epand et al. 2010; Wadhwani et al. 2012).

Trp localization studies of the 3 peptides showed that if a peptide is active against a certain membrane lipid, it exhibits a significant blue shift of the Trp emission wavelength upon interaction with the membrane indicating a more hydrophobic environment of Trp due to interaction with the membrane interface. In the case of the repeat sequence peptide this blue shift only occurs in presence of the target lipid PS present on the surface of cancer membranes, whereas in the presence of PC no blue shift appears, going in hand with a selective toxicity against cancer cells in vitro. The *N*-acylated peptide however reveals a blue shift in the presence of both model systems though the accessibility of Trp (K_sv_) is more highly decreased in the presence of the cancer mimic, again resembling activity in vitro, where 6-MO-LF11-322 is also slightly active against non-cancer cells. Also leakage experiments show that membrane permeabilizing specificity of 6-MO-LF11-322 for the cancer model system, which is threefold over the non-cancer model, resembles the relatively low threefold specificity for melanoma cells in vitro, a very good demonstration of the correlation of the model studies with biological activity (see Table 4).

Further information on the studied peptides was given by CD measurements and secondary structure predictions. The differences between the selective and non-selective peptides were quite obvious. Significant structural changes for LF11-322 and the repeat sequence peptide appear only in the presence of the negatively charged cancer mimic SDS. The *N*-acylated peptide again changes its structure in environment of both models correlating with its lower specificity. Although structural studies in the presence of SDS and DPC forming micelles cannot be absolutely compared to changes in the presence of a bilayer, the changes can be correlated to changes induced by the outer monolayer of the cancer or non-cancer cell membranes, which is the first and most important point of interaction. Strikingly only the *N*-acylated less selective peptide shows an increase of the α-helical content in the presence of both model systems, differently LF11-322 and R-DIM-P-LF11-322 show an increase of the β-sheet content but as mentioned only in presence of the cancer model SDS. According to a cluster analysis of Dennison et al. (2010) out of 158 amphipathic α-helical peptides ~80 % were shown to be toxic to both cancer and non-cancer cells. Such a tilted structure has also been associated already earlier with relatively non-specific means of cell membrane lysis (Hoskin and Ramamoorthy 2008). Interestingly however only 2 % of all antitumor peptides listed (Wang et al. 2009; Wang and Wang 2004) exhibit a β-sheet structure (Riedl et al. 2011b), though being quite successful in in vitro and in vivo studies. Antitumor peptides with β-hairpin structure are e.g., natural bovine lactoferricin (Hwang et al. 1998) or designed SVS-1 (Sinthuvanich et al. 2012).

From the differences in in vitro activity displayed by the peptide moiety and the repeat sequence peptide it was however surprising that both peptides show quite similar structural characteristics in solution and model system. Considering the shortness of LF11-322, it is even questionable that a β-sheet conformation, as proposed by CD data, is possible. Secondary structure prediction (PEP-FOLD, Fig. 6) performed for the short peptide LF11-322 indicates that the peptide forms two turns between Phe^2^ and Arg^6^. This is in agreement with previous NMR data that peptide LF11-322 forms a helical turn between residues 3-6 in the presence of SDS micelles (Zorko et al. 2009). However, maybe the peptide moiety is too short to form an as stable secondary structure as in comparison to R-DIM-P-LF11-322 which is predicted to adopt a β-hairpin structure. It is moreover reasonable that two peptide moiety stretches might arrange like a dimer (polymer) and thereby arrange in loose β-sheets stabilized by H-bonds. The repeat sequence peptide however is fixed in this conformation via peptide bond and is able to create stronger membrane perturbance and finally higher membrane permeabilization, which can explain its highly increased activity in model and cell system. This theory of an arrangement of LF11-322 like a polymer is maybe supported by the fact that the CD spectra of LF11-322 in solution and even more in presence of SDS exhibit a positive band at 230 nm. In literature such a band is described as a hallmark for a polyproline type II (PPII) helix (Woody 1992). Such structures are common in e.g. unfolded polypeptides (Shi et al. 2002). Nevertheless from model studies, LF11-322 could be reasoned to be more active than in vitro studies show. Minor activity in vitro could therefore also be due to minor stability of the peptide moiety considering that proteins with N-terminal Pro amongst others are described to exhibit short half-lives of half an hour (Westphal et al. 2003). Proteolytic instability could on the one hand be the reason for the low activity of the peptide moiety and proteolytic protection by structural conformation, on the other hand explain the highly increased activity of the repeat sequence R-DIM-P-LF11-322. A stable structure of bovine Lactoferrin has been reported before to be a prerequisite for antitumor even more than for antimicrobial activity (Bellamy et al. 1992; Schibli et al. 1999; Yang et al. 2004). To check stability we tested activity of LF11-322 in the presence of buffer and absence of serum and could not detect any improvement of activity after 1 h (data not shown). We chose such a short period to prevent membrane effects caused by growth deficiencies due to lack of serum components. However selective peptides that act over apoptosis rather than necrosis probably need longer time periods for killing, which might hinder the clear conclusion of this experiment. CD data of the *N*-acyl derivative 6-MO-LF11-322 show an increase of the α-helical proportion of the peptide in presence of SDS and DPC. 2D-NMR of a related lipopeptide, *N*-lauryl-LF11, indicated a more defined structure in presence of SDS than in DPC (Japelj et al. 2007). However, a defined structure of the peptide was shown to be stabilized by the *N*-acyl group, which might also have impact on the increased antitumor activity of 6-MO-LF11-322.

A proposed model for the interaction of the studied peptides with cancer membranes is the following: Peptides first are attracted to the cancer membrane by electrostatic interaction with PS (Gifford et al. 2005; Papo and Shai 2005). The fact that activity on WM164 was slightly weaker than activity against SBcl-2, though WM164 exposes higher levels of PS (~threefold) than SBcl-2 (Riedl et al. 2011a; Zweytick et al. 2011a), suggests that the degree of antitumor activity does not seem to go in hand with the degree of PS exposed. Probably just an elevated (threshold) level of PS exposed compared to non-cancer cells is required for antitumor activity. However other negative charges on the cancer surface as targets as exhibited by sialic acid residues, which appear to be linked to some glycoproteins (e.g. mucins) and glycolipids present on membrane surfaces and are partially overexpressed within certain cancer types, do not seem to interfere with the LF11 derived peptide activity, which was demonstrated by maintenance of activity after sialidase treatment (manuscript Riedl et al. in preparation). At a certain concentration the peptides once attracted to the negatively charged PS on the cancer membrane seem to penetrate with their hydrophobic amino acids like an anchor into the membrane. This can lead to direct (fast) membrane lysis, which in the case of the *N*-acylated peptide partially might occur, probably also due to its high hydrophobic moiety. This to some degree also causes interaction with neutral lipids on cancer and as well on non-cancer membranes. The repeat sequence however can due to its adoption of a defined structure only in the presence of PS specifically interact with cancer membranes. Probably it enters the membrane selectively over the PS key and then interacts with inner lipid targets causing apoptosis. This idea is also supported by the fact that the repeat sequence is acting more slowly than the *N*-acylated peptide (data not shown), which lets preferentially assume a killing mechanism via apoptosis.

In our study we could prove that quite low hydrophobicity but a prolonged sequence combined with a higher net positive charge are sufficient or needed to improve the antitumor activity of hLFcin derivatives. Conformational changes exclusively in the presence of the target membrane seem to induce selective activity. As can be seen by the *N*-acylated peptide improvement of activity by increase of the hydrophobic moiety also might implicate increased effect on neutral membrane components and therefore also provoke toxicity against healthy non-cancer cells. Adoption of a stable secondary structure however seems to be possible within the repeat sequence peptide R-DIM-P-LF11-322 compared to the short peptide LF11-322 resulting in high and selective toxicity on cancer cells in vitro. In conclusion, we were able to demonstrate a significant correlation between the toxicity of the studied peptides against human cancer or non-cancer cells and interaction with the respective model membrane systems, proving PS as a molecular target of the peptides on cancer cells. Such a specific marker offers the possibility of treatment of all cancer types, also with so far poor treatability, and even metastases with minimization of side effects and formation of resistance.
